# BAG3 and HIF-1**α** Coexpression Detected by Immunohistochemistry Correlated with Prognosis in Hepatocellular Carcinoma after Liver Transplantation

**DOI:** 10.1155/2014/516518

**Published:** 2014-05-08

**Authors:** Heng Xiao, Rongliang Tong, Shaobing Cheng, Zhen Lv, Chaofeng Ding, Chengli Du, Haiyang Xie, Lin Zhou, Jian Wu, Shusen Zheng

**Affiliations:** ^1^Division of Hepatobiliary and Pancreatic Surgery, Department of Surgery, First Affiliated Hospital, Zhejiang University School of Medicine, Hangzhou 310003, China; ^2^Key Lab of Combined Multi-Organ Transplantation, Ministry of Public Health, Hangzhou 310003, China

## Abstract

*Objective*. The objective is to determine the effects of BAG3 and HIF-1**α** expression on the prognosis of HCC patients after liver transplantation. *Methods*. Samples from 31 patients with HCC receiving liver transplantation were collected for this study. The immunohistochemistry was used to detect the expression of BAG3 and HIF-1**α** of HCC samples. *Results*. According to the immunohistochemistry results, BAG3 and HIF-1**α** staining were significantly associated with tumor TNM stage (*P* = 0.004, *P* = 0.012). A significant association between high BAG3/HIF-1**α** levels and a shorter overall survival was detected, so as the combined BAG3 and HIF-1**α** analysis. *Conclusion*. The results suggested that the expression level of BAG3 and HIF-1**α** is efficient prognostic parameters in patients with HCC after liver transplantation.

## 1. Introduction

Hepatocellular carcinoma (HCC), a highly vascular tumor, is the third leading cause of cancer death worldwide and the second in China [1, 2]. Because existing therapies are insufficient for the high frequency of tumor recurrence after liver transplantation, the prognosis of HCC patients remains pessimistic. So it is important to establish the identity of new targets for therapeutic approach that will improve the prognosis of HCC patients after liver transplantation.

BAG3 can interact with different partners through a BAG domain, a WW domain, and a proline-rich repeat [3]. As a multifaceted protein, BAG3 regulates many biological processes and impacts the progression of tumor in different ways. The expression of BAG3 was reported that related to survival, apoptosis, motility and adhesion, angiogenesis, and epithelial-mesenchymal transition of human neoplastic cells [3–9]. In most studies, BAG3 is demonstrated as a protein favoring tumor progression.

In our previous research, a decreased expression of HIF-1*α* was observed in knockdown of BAG3 by western blot [10]. As an important regulator in hypoxia adaptation, HIF-1*α* regulates proliferation, apoptosis, metastasis, inflammation, and angiogenesis in tumors [11, 12]. In a report of Dai et al., HIF-1*α* was found to affect the inflammation and angiogenesis of HCC. The expression level of HIF-1*α* was also observed associated with the development and prognosis of HCC in that study [13]. The value of HIF-1*α* in predicting prognosis of HCC was supported by some researchers [14–16]. However, there are also evidences that object to it [17, 18].

In the present work we have evaluated the expression of BAG3 and HIF-1*α* in HCC tissue and analyzed the prognosis of HCC after liver transplantation and we hope to find some new molecular targets that will improve the prognosis of HCC patients after liver transplantation.

## 2. Materials and Methods

### 2.1. Cell Culture

Eight human HCC cell lines (HepG2, Huh-7, Bel-7402, SK-Hep-1, SMMC-7721, MHCC-97L, MHCC-97H, and MHCC-LM3) and one immortalized liver cell lines (L-02) were purchased from Cell Bank of Type Culture Collection of Chinese Academy of Sciences, Shanghai Institute of Cell Biology, Chinese Academy of Sciences, and were cultivated as described by the suppliers. Cell lines treatment was as follows: hypoxia (O_2_ of 1%) in a hypoxia chamber for 72 hours or add CoCl_2_ (300 *μ*mol/L) to the medium to induce hypoxia condition.

### 2.2. Western Blot and RT-PCR

Western blot and RT-PCR were performed as described previously [10].

### 2.3. Study Subjects

Samples from 40 patients with HCC receiving liver transplantation at our hospital (First Affiliated Hospital, Zhejiang University School of Medicine, Zhejiang, China) between 2005 and 2010 were collected for this study. Letters of consent were obtained from all patients, and the experimental protocols were approved by the local ethics committee. Patient charts were reviewed to obtain clinical data about age, gender, tumor size, AFP, HBsAg, vascular invasion, TNM stage (AJCC), tumor differentiation, TACE and RFA usage before LT, Rapamycin usage after LT, time of recurrence, and death or time of last followup. Patient survival was calculated from the day of surgery until death, in months. However, 5 patients had cholangiocarcinoma, and 4 patients lost to followup. So we just analyzed 31 patients at last.

### 2.4. Immunohistochemistry

The paraffin-embedded tissue as that used for the HE-stained section was chosen for immunohistochemistry. They were cut at 3 um, deparaffinized in xylene, and rehydrated in a series of graded alcohol dilutions. Heat epitope retrieval was done for 20 minutes in target-retrieval solution at pH 7.5. Sections were incubated with a rabbit monoclonal antibody to human BAG3 (cat. number: 2783-1, Epitomics-an abcam company, Cambridge, MA, USA; dilution 1/1000) at dilution of 1 : 100 and with a rabbit monoclonal antibody to human HIF-1*α* (cat. number: 2015-1, Epitomics-an abcam company, Cambridge, MA, USA; dilution 1/1000) at dilution of 1 : 200 overnight at 4C°. Slides were then incubated with HRP at room temperature for 30 minutes and were visualized using DAB as chromogen for 5–10 minutes.

Sections were scored semiquantitatively as follows [19]: (negative), 0% immunoreactive cells; +≦5% immunoreactive cells; ++ >5–50% immunoreactive cells; +++≧50 immunoreactive cells. For statistical purposes, cases with scores 0 and + were considered low expression and those with scores ++ and +++ were considered high expression.

### 2.5. Statistical Analysis

The data were performed using SPSS version 17.0. The chi-square test or Fisher's exact test was used to evaluate any potential association between the BAG3/HIF-1*α* expression and the clinicopathologic parameters. Overall survival and tumor-free survival rates were calculated with the Kaplan-Meier method, and the statistical difference between survival curves was determined with the log-rank test. Statistical significance was accepted if *P* < 0.05.

## 3. Results

### 3.1. Clinicopathologic Characteristics of Patients Included in the Study

The study included tumors from 31 patients (28 males and 3 females). Patients' characteristics are shown in Table 1. BAG3 staining was significantly associated with tumor size (*P* = 0.027) and tumor TNM stage (*P* = 0.004). Like BAG3, HIF-1*α* staining was significantly associated with tumor TNM stage (*P* = 0.012), but not with tumor size (*P* = 0.22). High expression of BAG3 and HIF-1*α* assessed was found as follows: BAG3 in 17 (54.8%) cases and HIF-1*α* in 18 (58.1%) cases.

### 3.2. BAG3 and HIF-1*α* Expression in the HCC Cell Lines

**Figure 1:**
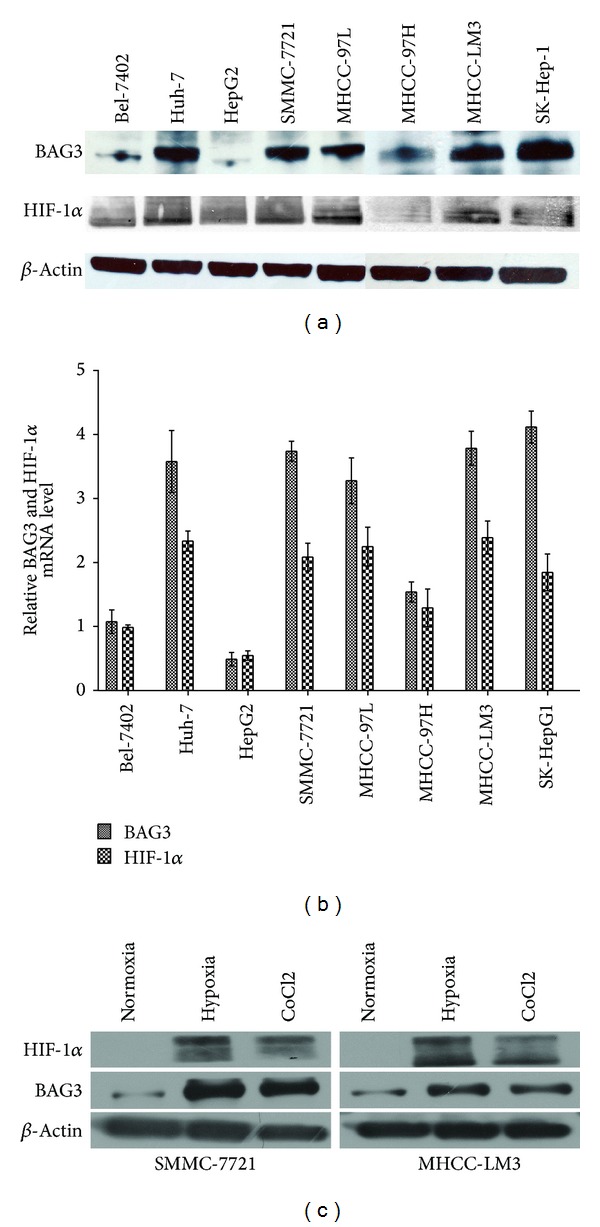
(a) BAG3 and HIF-1*α* expressions were evaluated in the indicated cell lines by western blot. (b) BAG3 and HIF-1*α* expressions were evaluated in the indicated cell lines by RT-PCR. (c) BAG3 and HIF-1*α* expressions of HCC cell lines under hypoxia were measured using western blot.

To prove the significance of the above clinical data, we examined the BAG3 and HIF-1*α* expression in the eight HCC cell lines. We found that HIF-1*α* showed the same change with the level of BAG3 expression (Figures 1(a) and 1(b)). To determine the relationship between BAG3 and HIF-1*α*, SMMC-7721 and MHCC-LM3 were cultured under the hypoxia condition. As shown in Figure 1(c), both BAG3 and HIF-1*α* expressions were increased under hypoxia condition.

### 3.3. Survival Analysis in Patients with HCC after Liver Transplantation

**Figure 2:**
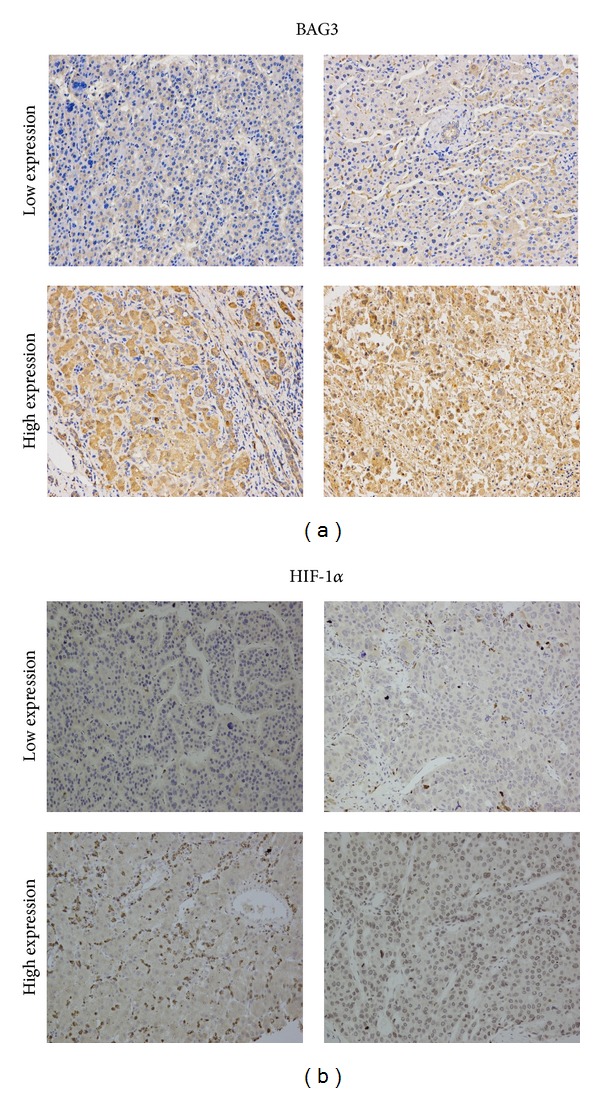
HCC samples were immunostained with BAG3 and HIF-1*α* antibody (×200). (a) Protein expression of BAG3 (up, low-expression; down, high-expression). (b) Protein expression of HIF-1*α* (up, low-expression; down, high-expression).

A low and high staining reaction of BAG3 in patients with HCC is shown in Figure 2(a). The 5-year overall survival rate for patients with low expression of BAG3 and for patients with high expression of BAG3 was 52.4% and 23.5% (*P* = 0.021), respectively. The 5-year tumor-free survival rate for patients with low expression of BAG3 and for patients with high expression of BAG3 was 62.3% and 25.0% (*P* = 0.183), respectively (Table 2).

A staining reaction of HIF-1*α* in patients with HCC is shown in Figure 2(b) like BAG3 (51.3% versus 27.8%, *P* = 0.013), but not tumor-free survival rate (*P* = 0.613) (Table 3).

**Figure 3:**
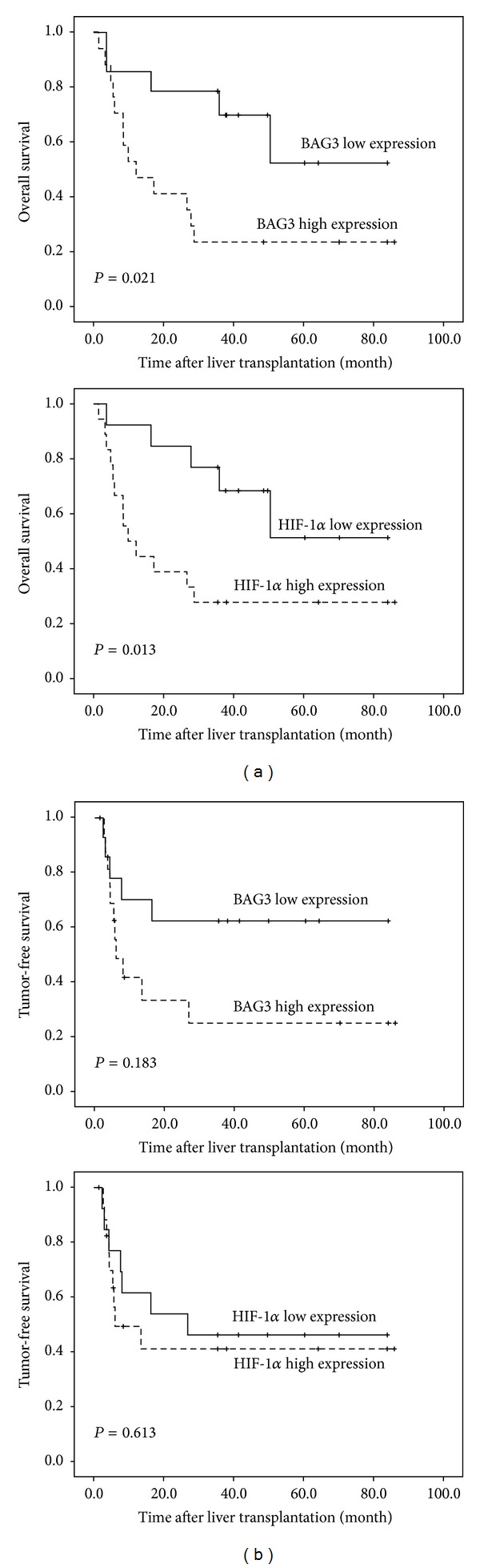
(a) BAG3 and HIF-1*α* overall survival rate. (b) BAG3 and HIF-1*α* tumor-free survival rate.

A Kaplan-Meier curve regarding the association between BAG3 and HIF-1*α* staining and overall and tumor-free survival is shown in Figure 3.

### 3.4. Combined BAG3 and HIF-1*α* Analysis

**Figure 4:**
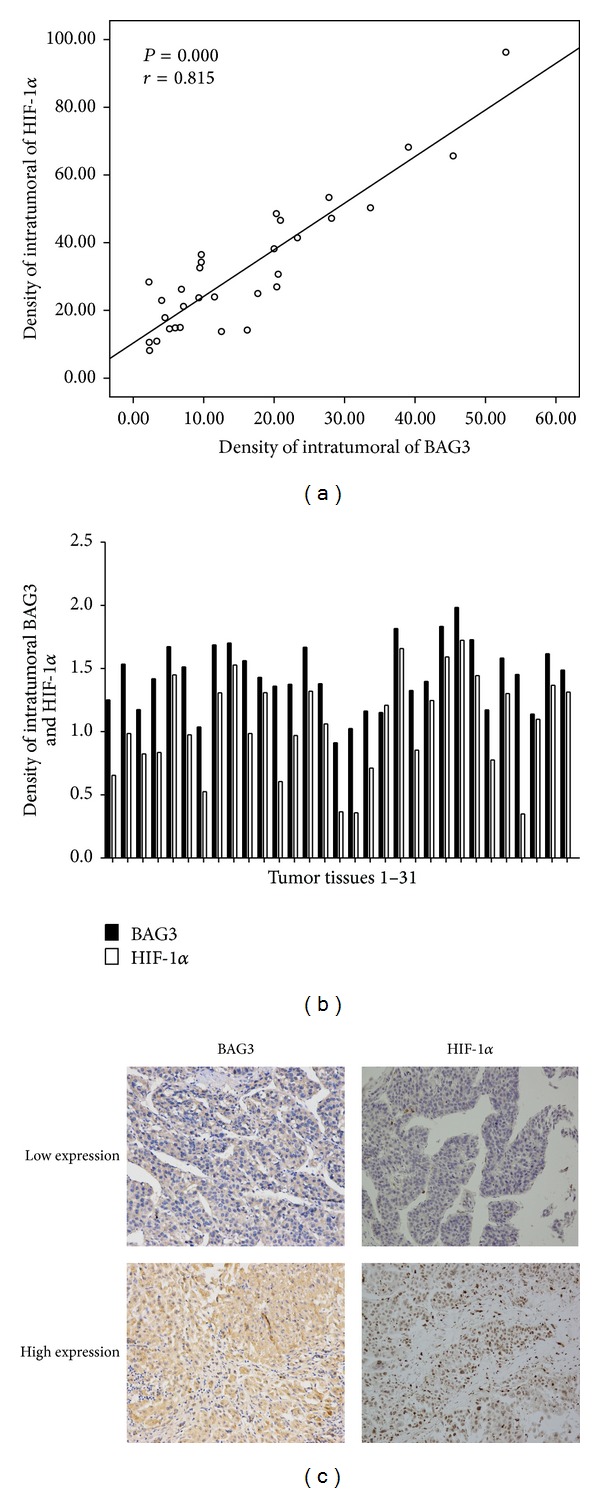
(a) Correlation between BAG3 expression and HIF-1*α* level was examined in tumor tissue derived from 31 patients. (b) Protein levels of BAG3 and HIF-1*α* were determined in 31 HCC samples. (c) Representative serial sections (one sample) of HCC immunostained for BAG3 and HIF-1*α* (×200).

**Figure 5:**
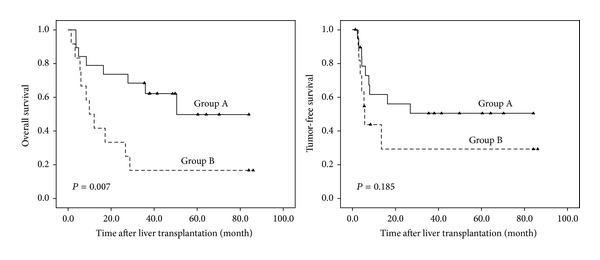
Overall survival and tumor-free survival rate of patients with HCC after liver transplantation in relation to coexpression of BAG3 and HIF-1*α*. Group A tumors had both BAG3 and HIF-1*α* low expression levels; group B had both BAG3 and HIF-1*α* high expression levels.

The frequency of BAG3 and HIF-1*α* staining in HCC is shown in Table 4. A significant correlation was observed between BAG3 and HIF-1*α* staining (*P* = 0.009). Tissue analysis revealed a correlation of BAG3 with HIF-1*α* (*r* = 0.815, *P* = 0.000), and HIF-1*α* showed the same change with the level of BAG3 expression in most of the tumor tissue (Figures 4(a) and 4(b)). Tumors were divided into two groups according to the BAG3 and HIF-1*α* expression (Figure 4(c)). Group A (*n* = 13) tumors had both BAG3 and HIF-1*α* low expression levels; group B (*n* = 12) had both BAG3 and HIF-1*α* high expression levels. Patients in group A had either worse overall survival or shorter tumor-free survival rate than group B (Figure 5) (*P* = 0.007, *P* = 0.185, resp.). Consistently, the 1-year and 5-year overall survival and tumor-free survival rate after liver transplantation were better for group A than group B (Table 5).

## 4. Discussion

BAG3 was found highly expressed, compared with normal human cells or tissue, in many solid tumors, such as HCC [10], nonsmall cell lung cancer [8], thyroid carcinoma [9], pancreatic cancer [20], glioblastoma [21], and colorectal carcinomas [22]. In most studies, BAG3 is demonstrated as a protein favoring tumor progression. However, there are also some opposite evidences. In a recently study, De-Hui Kong et al. observed a role of BAG3 in preventing antiapoptotic effect [23]. Li et al. reported that BAG3 could stabilize JunD mRNA to promote growth inhibition mediated by serum starvation [24]. In addition, a previous research also supported the antiproliferative function of BAG3 in human promyelocytic leukemia HL-60 cells [25]. However, a high expression of BAG3 is not equal to poor prognosis. With the results of an immunohistochemical study of prostate carcinoma, the expression level of BAG3 was observed to progressively increase from low- to well-differentiated carcinoma. That study also demonstrated that a low expression level of BAG3 predicted a poor prognosis of patients with prostate carcinoma [19]. As a conclusion, the role of BAG3 in tumors is not that clear.

A recent research by us focused on the role of BAG3 in HCC and demonstrated that BAG3 promoted epithelial–mesenchymal transition, tumor growth, invasiveness, and angiogenesis of HCC [10]. In that study, we also found that the HIF-1*α* signaling pathway might be included in the mechanisms of BAG3 in regulating the metastasis and angiogenesis of HCC cells. However, the conjecture has not been proved yet.

With the observations above, we evaluated the expression of BAG3 and HIF-1*α* in HCC cell lines by western blot and RT-PCR; we found that HIF-1*α* showed the same change with the level of BAG3 expression. Meanwhile, we detected the BAG3 and HIF-1*α* expression in HCC tissue by immunohistochemistry and tried to observe their role in predicting the prognosis of patients who were diagnosed as HCC and received liver transplantation. The high expression of BAG3 and the high expression of HIF-1*α* are both demonstrated relating to later TNM stage and poor overall survival. Compared with HIF-1*α*, BAG3 shows a better correlation with tumor size. In addition, our study is the first to demonstrate the value of BAG3 and HIF-1*α* in predicting the prognosis of HCC in the field of liver transplantation.

In this study, we also observed the positive correlation between the expression of BAG3 and HIF-1*α* in HCC. And when HCC cell lines were cultured under hypoxia condition, we found that both HIF-1*α* and BAG3 protein levels were significantly increased. Consistent with this result, a decreased expression of HIF-1*α* was observed in knockdown of BAG3 by western blot in our previous research [10]. Furthermore, HIF-1*α* was reported that could bind to and transcriptional upregulate HSP70-2 (also known as heat shock 70 kDa protein 1B), and BAG3 protein is a cochaperone that interacts with the ATPase domain of Hsp 70 [3, 26]. All of these evidences indicated that the mechanisms of BAG3 in impacting the progression of tumor may involve the HIF-1*α* signaling pathway. However, further studies and more evidences are necessary to make the mechanism clear.

The limitations of our study are listed as follows. Firstly, a significant correlation between tumor-free survival and the expression level of BAG3 and/or HIF-1*α* is not observed. This may be a result of the low number of patients. Second, an effective statistical analysis of the patients with BAG3 high-/HIF-1*α* low- or BAG3 low-/HIF-1*α* high-expression in tumor is not available. The lacking of patients could be the reason for both. Furthermore, our study is retrospective. And the interrelationship between BAG3 and HIF-1*α* needs further studies.

## 5. Conclusions

Our study is the first to demonstrate that the expression level of BAG3 and HIF-1*α* is efficient prognostic parameters in patients with HCC after liver transplantation.

## Figures and Tables

**Table 1:** Relationship between BAG3/HIF-1*α* expression and clinicopathologic features.

Variable	BAG3 density		*P* value	HIF-1*α* density		*P* value
	Low-BAG3	High-BAG3		Low-HIF-1*α*	High-HIF-1*α*	
In general						
Tumor tissue	14	17		13	18	
Sex						
Male	12	16	0.425	12	16	0.624
Female	2	1		1	2	
Age (years)						
≦50	8	9	0.551	9	8	0.158
>50	6	8		4	10	
Tumor size (cm)						
≦5	9	4	0.027*	7	6	0.22
>5	5	13		6	12	
AFP (ng/mL)						
≦400	8	12	0.343	8	12	0.343
>400	6	5		5	6	
HBsAg						
Positive	12	14	0.597	12	14	0.285
Negative	2	3		1	4	
Anti-HCV						
Positive	1	1	0.708	1	1	0.671
Negative	13	16		12	17	
Vascular invasion						
Yes	6	12	0.117	6	12	0.22
No	8	5		7	6	
TNM stage						
I-II	10	3	0.004*	9	4	0.012*
III-IV	4	14		4	14	
Tumor differentiation						
I-II	2	3	0.597	2	3	0.659
III-IV	12	14		11	15	
TACE or RFA using before LT						
Yes	5	7	0.525	4	8	0.347
No	9	10		9	10	
Rapamycin using after LT						
Yes	2	4	0.429	2	4	0.501
No	12	13		11	14	

AFP: *α*-fetoprotein. TACE: transcatheter arterial chemoembolization. RFA: radiofrequency ablation.

*P < 0.05.

**Table 2:** Relationship between BAG3 expression and survival rate.

Survival measurement	BAG3 density		*P* value
	low-BAG3	high-BAG3	
1-year overall survival (%)	85.7 ± 9.4	52.9 ± 12.1	0.021*
5-year overall survival (%)	52.4 ± 17.9	23.5 ± 10.3	
1-year tumor-free survival (%)	70.1 ± 12.6	41.7 ± 12.7	0.183
5-year tumor-free survival (%)	62.3 ± 13.4	25.0 ± 11.9	

*P < 0.05.

**Table 3:** Relationship between HIF-1*α* expression and survival rate.

Survival measurement	HIF-1*α* density		*P* value
	low-HIF-1*α*	high-HIF-1*α*	
1-year overall survival (%)	92.3 ± 7.4	50.0 ± 11.8	0.013*
5-year overall survival (%)	51.3 ± 17.8	27.8 ± 10.6	
1-year tumor-free survival (%)	61.5 ± 13.5	49.3 ± 12.8	0.613
5-year tumor-free survival (%)	46.2 ± 13.8	41.1 ± 13.0	

*P < 0.05.

**Table 4:** Correlation of BAG3 and HIF-1*α* in HCC.

HIF-1*α*	BAG3			
	Negative	+	++	+++
Negative	0	4	0	0
+	0	6	2	1
++	0	4	0	4
+++	0	0	1	9

Kendall's correlation coefficient, *P* = 0.009.

**Table 5:** Relationship between HIF-1*α* and BAG3 coexpression and survival rate.

Survival measurement	BAG3/HIF-1*α* density		*P* value
	low-BAG3/HIF-1*α*	high-BAG3/HIF-1*α*	
1-year overall survival (%)	90.0 ± 9.5	42.9 ± 13.2	0.007*
5-year overall survival (%)	45.7 ± 21.2	14.3 ± 9.4	
1-year tumor-free survival (%)	60.0 ± 15.5	35.9 ± 13.9	0.185
5-year tumor-free survival (%)	50.0 ± 15.8	23.9 ± 13.5	

*P < 0.05.
